# The Effectiveness of Physical Therapy Intervention in a Seven-Year-Old Child With Congenital Talipes Equinovarus: A Case Report

**DOI:** 10.7759/cureus.48423

**Published:** 2023-11-07

**Authors:** Spandan Munjewar, Kamya J Somaiya, Manali A Boob, Pratik Phansopkar

**Affiliations:** 1 Musculoskeletal Physiotherapy, Ravi Nair Physiotherapy College, Datta Meghe Institute of Higher Education and Research, Wardha, IND

**Keywords:** musculoskeletal disorder, physical therapy, congenital talipes equinovarus, clubfoot, cerebral palsy

## Abstract

Congenital talipes equinovarus (CTEV), a prevalent congenital anomaly, is characterized by the backward bending of the hindfoot, the inward turning of the midfoot, and the turning or tilting of the front foot. The likelihood of experiencing clubfoot is higher in males and among firstborn offspring. Both genetic and environmental elements are recognized as factors that play a role in the occurrence of this developmental irregularity. It is diagnosed clinically because the abnormality has been visible since childhood, where one or both feet point downward and inward. If the deformity is not addressed, tarsal bones and joints will stiffen over time, further causing an inability to walk and stand, causing additional limb deformities. Late presentations are typical in less developed nations because of a lack of awareness, access to care, or a holdup in referral. We have a case of a seven-year-old spastic cerebral palsy (CP) male child with congenital talipes equinovarus. While assessing, we found visible deformities at the knee and ankle joints. Wedge osteotomy and Achilles tendon lengthening surgery were performed. Probably, extensive soft tissue surgery is the best option for treating clubfoot. A physical therapist may use stretching, proprioceptive neuromuscular facilitation (PNF), joint mobilization, and joint compression to enhance the foot's alignment, mobility, and range of motion (ROM) to keep the joint in the correct position. Physical therapy greatly reduced stiffness. The physiotherapy treatment plan we used was highly beneficial in enhancing the patient's quality of life, increasing his level of independence, and enhancing his participation in his activities of daily living (ADLs).

## Introduction

Congenital talipes equinovarus (CTEV) is a common congenital condition characterized by hindfoot equinus, midfoot varus, and forefoot supination and adduction, with an incidence of one per 1,000 live births. If neglected, it will lead to callus formation, further causing discomfort, deformity, and long-term impairment. Conservative (such as stretching or splinting) or surgical interventions would both be feasible [1]. There are chances of reoccurrence until the child turns 6-7 years old [2]. Its etiopathogenesis and inheritance patterns of the defect are not fully known [3]. A combination of environmental and genetic factors are known contributors to this developmental abnormality [4]. The majority of CTEV cases are idiopathic and present as a single-birth abnormality. By researching the known causes of this birth abnormality, it may be possible to better understand the pathophysiological basis of idiopathic CTEV [5]. Children with neuromuscular conditions such as cerebral palsy (CP), a disorder characterized by abnormal posture, movement, and tone, with an incidence of 2-3 per 1,000 live births [6], were found to exhibit equinus in 61% of cases [7]. Equinus is the inability to dorsiflex the foot above plantar flexion while maintaining a neutral position for the hindfoot and an extended knee [8]. Equinovarus can occur in one or both feet, and it is more common in boys than in girls [9]. The tibialis posterior and tibialis anterior are two muscles involved in varus deformity [10].

Negligence due to poverty, social stigma, a lack of proper healthcare services, and a lack of education interferes with the early presentation of a child with CTEV, and management becomes even more challenging in older children [11]. Correcting equinus and equinovarus abnormalities in CP may improve walking function. Young children should be managed nonoperatively. These abnormalities are best treated with muscle-balancing techniques such as split tibialis anterior tendon transfer (SPLATT), Achilles tendon lengthening, and gastrocnemius aponeurosis lengthening [12]. Although malformations such as foot deformities are very common among children and adults who do not have CP, these disorders tend to have a greater negative impact on children with CP. Since children with CP frequently have stiff muscles, it can be more challenging for them to adjust to foot deformities. Treatment is usually required to address the issue because it frequently impacts the child's mobility and gait. In developing countries, children suffering from clubfoot with cerebral palsy need orthopedic assistance [13]. We present a case of a seven-year-old male child with spastic cerebral palsy. Clubfoot developed later, secondary to spastic cerebral palsy. A physical therapist may use stretching, PNF, joint mobilization, and joint compression to enhance the foot's alignment, mobility, and range of motion (ROM) to keep the joint in the correct position. Physical therapy greatly reduced stiffness. The physiotherapy treatment plan we used was highly beneficial in enhancing the patient's quality of life, increasing his level of independence, and enhancing his participation in his activities of daily living (ADLs).

## Case presentation

Patient information

**Figure 1:**
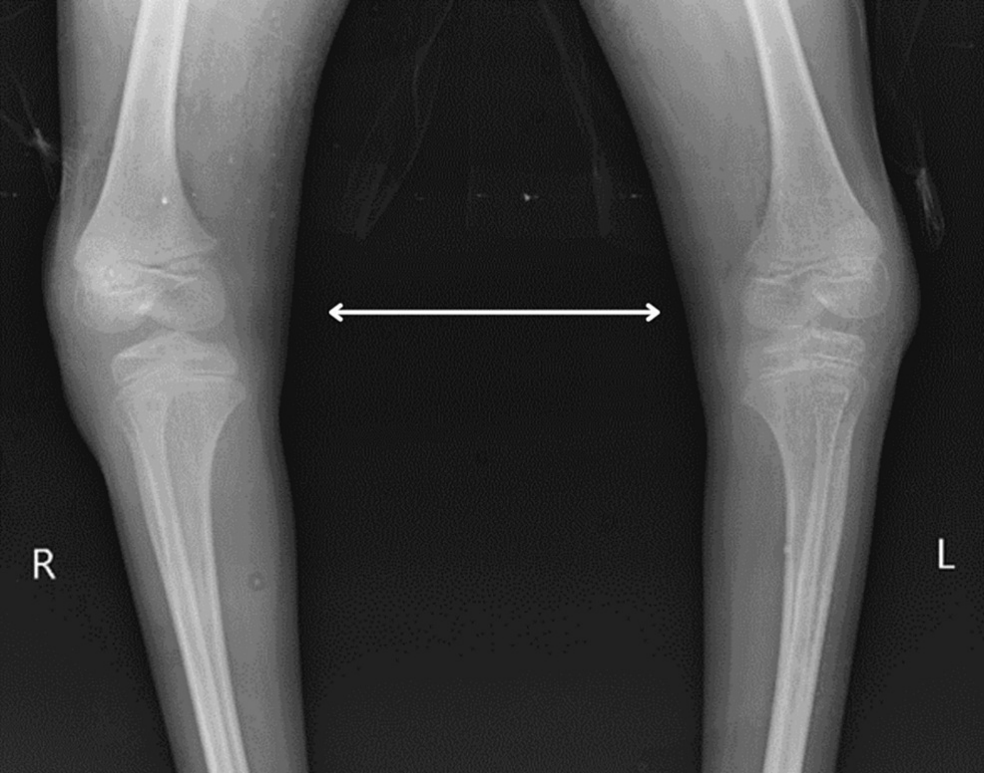
X-ray showing the leg bowing outward at the knee, while the lower leg is angled medially

**Figure 2:**
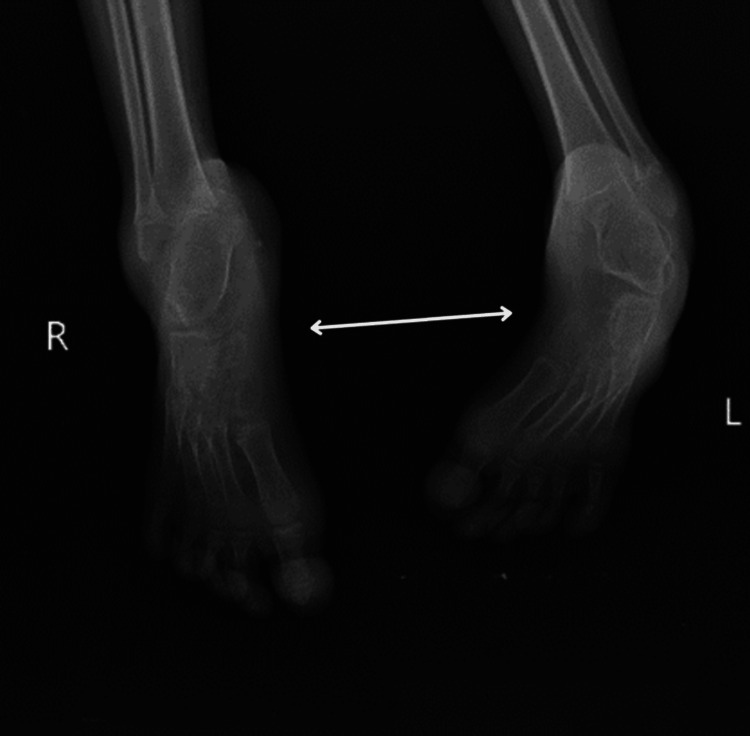
X-ray showing visible deformity at the ankle joint where the foot is in fixed plantar flexion, the longitudinal arch of the foot is exaggerated, and the foot is inverted and adducted at the midtarsal joint

A seven-year-old male child (who weighed 2 kg at birth) was brought with an inability to stand and walk independently and deformity of both lower limbs since birth, which was insidious in onset and gradually progressive. Developmental milestones were delayed. For the above complaints, the patient went to a local practitioner, where an X-ray was done, which suggested a reduced talocalcaneal and talometatarsal angle (Figures 1, 2). He was managed with casts, which was ineffective. Due to persistent difficulty walking, the child was brought to Acharya Vinoba Bhave Rural Hospital (AVBRH). Here, investigations were done, which showed that the foot is in fixed plantar flexion, the longitudinal arch of the foot is exaggerated, and the foot is inverted and adducted at the midtarsal joint, and he was diagnosed with CTEV. As a result, the child has undergone a surgical procedure for bilateral Achilles tendon lengthening and dorsolateral wedge resection with a bilateral cast above on August 20, 2023. Presently, the patient complains of difficulty walking and pain in the heel. The family history was not significant. Additionally, there was a documented history of reduced appetite and weight loss. He did not present any known comorbidities or complaints related to bladder or bowel function. The patient was referred to the physiotherapy department for further management.

Clinical findings

The patient was examined in a supine position with both the anterior superior iliac spine (ASIS) at the same level. Calf muscle wasting with visible deformity at the knee and ankle was appreciated on observation. On palpation and movement examination, there was no tenderness present, the hip and knee were in a flexed position, and the patella was facing outward at 5 degrees. The plumb line test was positive as ankle joint cavus, varus, and equinus deformities are present. The range of motion (ROM) in the knee joint is restricted. According to the Modified Ashworth Scale (MAS), grade 3 spasticity is present at ankle plantar flexors.

Physiotherapy interventions

Proper physiotherapy intervention was given for four weeks as outlined in Table 1.

**Table 1:** Physiotherapy intervention ROM: range of motion, NA: not applicable, DB: Denis Brown, CTEV: congenital talipes equinovarus

Goals	Physiotherapy intervention	Dosage/frequency	Rationale
Reduce pain	Icing over the ankle joint	For 7 minutes, thrice daily	Ice helps reduce the pain by numbing the area.
Relieve muscle tightness and contracture	Sustained stretching of plantar flexors and dorsiflexors muscles	For 45 seconds, every four hours	Muscles will be gradually lengthened through a joint's full ROM; the final position should be held for a few seconds.
	CTEV shoes and DB splint	14 hours/day	
		NA	It maintains the correct foot position.
Provide proprioceptive sensation	Proprioceptive neuromuscular facilitation of ankle joint (technique used: hold-relax)	Thrice daily	It will increase the ROM by increasing the length of the muscle and further increasing neuromuscular efficiency.
Achieve bed mobility	Mat exercises	Twice daily	It increases mobility and functional independence.
Obtain normal ROM	Joint compression at the ankle joint	Thrice daily	It is helpful in gaining a sense of proprioception in the initial stages.
Gait training	Ambulation with a walker	NA	This aims to make the child ambulatory in his activities of daily living.

Follow-up and outcomes

The outcome measures we utilized to assess the effectiveness of our intervention are mentioned in Tables 2-4.

**Table 2:** Outcome measures pre- and post-treatment scores Bangla clubfoot score: 85%-100%: very good, 70%-85%: good, 60%-70%: fair, <50%: poor SF-36: The lower the score, the higher the disability (range: 0-100). SF-36: short-form 36

Outcome measures	Pre-treatment score	Post-treatment score
Bangla clubfoot score	65/100	80/100
SF-36 quality of life scale	35/100	65/100

**Table 3:** Range of motion of the lower limb Normal range values: knee flexion: 0°-135°, knee extension: 135°-0°, ankle plantar flexion: 0°-50°, ankle dorsiflexion: 0°-20°, inversion: 0°-35°, eversion: 0°-30° AROM: active range of motion

Movement	Pre-treatment AROM		Post-treatment AROM	
	Right	Left	Right	Left
Knee flexion	0°-95°	0°-100°	0°-120°	0°-130°
Knee extension	95°-0°	100°-0°	120°-0°	130°-0°
Plantar flexion	0°-30°	0°-25°	0°-40°	0°-40°
Dorsiflexion	0°-5°	0°-10°	0°-15°	0°-20°
Inversion	0°-20°	0°-20°	0°-30°	0°-30°
Eversion	0°-5°	0°-10°	0°-15°	0°-15°

**Table 4:** Manual muscle testing for the lower limb (graded using the MOMMT system) 0: no contraction, 1: flickering contraction, 2: full ROM with gravity eliminated, 3: full ROM against gravity, 4: full ROM against gravity, moderate resistance, 5: full ROM against gravity, maximal resistance MOMMT: Modified Oxford Manual Muscle Testing, ROM: range of motion

Movements of the right lower limb	Pre-treatment MOMMT grades		Post-treatment MOMMT grades	
	Right	Left	Right	Left
Ankle plantar flexion	3/5	4/5	3/5	4/5
Ankle dorsiflexion	2/5	3/5	3/5	4/5
Inversion	3/5	4/5	4/5	4/5
Eversion	2/5	3/5	3/5	4/5

## Discussion

Various conservative techniques such as the kite method, the Ponseti technique, and the French method have shown varying degrees of success. Balance, coordination, gross motor function, strength, and agility issues may occur in patients with clubfeet. However, when conservative management fails, then surgical procedure becomes the option. It is important to consider neurological developmental issues at the time of assessment. Patients with cerebral palsy who have equinovarus foot deformity should undergo Achilles tendon lengthening to correct it when they present late for treatment [14].

The common surgical techniques for CTEV include posteromedial soft tissue release and Achilles tendon elongation. The immediate results of these operations seem excellent. However, due to a lack of postoperative physiotherapy management and follow-up, long-term results are not very lasting. Therefore, postoperative physiotherapy plays a vital role in the prevention of secondary complications and the persistence of results [15].

According to Mishra et al., a physiotherapy approach that includes strengthening, stretching, and taping has been demonstrated to be useful in addressing congenital talipes equinovarus (CTEV) [16]. The case study carried out by Ezeukwu and Maduagwu suggests that treating CTEV as soon as possible after delivery might aid in excellent recovery and decrease treatment costs while alleviating the psychological strain on both the patient's parents and care provider [17]. A tendo-Achilles lengthening (TAL) is routinely performed in the clinic [18]. Before and after surgery, voluntary and automatic muscle activities were compared, and it was discovered that fresh muscle activation was still possible [19].

Physical therapy substantially reduced stiffness and made surgical treatment considerably simple [20]. There is a need to initiate physiotherapy treatment for CTEV patients as soon as possible. Our study's primary objective was to develop a regimen for CTEV patients, and satisfactorily, the physiotherapy plan we used was quite successful in improving the patient's quality of life, level of independence, and participation in his activities of daily living (ADLs). Clinically, this will be very helpful in treating this population.

## Conclusions

Congenital talipes equinovarus is a musculoskeletal disorder that when presented late, the foot cannot move up and down as it normally would, the muscles get stiffened, and patients may experience pain later in life. In this case, the patient is unable to walk/stand, and there is a deformity in both limbs. Physiotherapy began immediately post-surgery to achieve early mobilization. The goal of physiotherapy is to restore the patient's maximum functional ability in using the lower limb, especially the foot. The physiotherapy treatment plan we used was highly beneficial in enhancing the patient's quality of life, increasing his level of independence, and enhancing his participation in his ADLs.
